# Impact of Extended Membrane Rupture on Neonatal Inflammatory Responses and Composite Neonatal Outcomes in Early-Preterm Neonates—A Prospective Study

**DOI:** 10.3390/diagnostics15020213

**Published:** 2025-01-18

**Authors:** Maura-Adelina Hincu, Liliana Gheorghe, Luminita Paduraru, Daniela-Cristina Dimitriu, Anamaria Harabor, Ingrid-Andrada Vasilache, Iustina Solomon-Condriuc, Alexandru Carauleanu, Ioana Sadiye Scripcariu, Dragos Nemescu

**Affiliations:** 1 Department of Mother and Child Care, University of Medicine and Pharmacy “Grigore T. Popa”, 700115 Iasi, Romania; maurahancu@gmail.com (M.-A.H.); iustina_condriuc@yahoo.com (I.S.-C.);; 2 Surgical Department, Faculty of Medicine, University of Medicine and Pharmacy “Grigore T. Popa”, 700115 Iasi, Romania; 3 Department of Morpho-Functional Sciences II, University of Medicine and Pharmacy “Grigore T. Popa”, 700115 Iasi, Romania; 4 Clinical and Surgical Department, Faculty of Medicine and Pharmacy, ‘Dunarea de Jos’ University, 800216 Galati, Romania

**Keywords:** prolonged rupture of membranes, inflammatory markers, adverse composite outcomes, early-preterm neonates

## Abstract

**Background/Objectives**: Prolonged prelabour rupture of membranes (PROMs), and the resulting inflammatory response, can contribute to the occurrence of adverse neonatal outcomes, especially for early-preterm neonates. This prospective study aimed to measure neonates’ inflammatory markers in the first 72 h of life based on ROM duration. The second aim was to examine the relationship between PROMs, serum inflammatory markers, and composite adverse neonatal outcomes after controlling for gestational age (GA). **Methods**: Data from 1026 patients were analyzed considering the following groups: group 1 (ROM < 18 h, *n* = 447 patients) and group 2 (ROM > 18 h, *n* = 579 patients). These groups were further segregated depending on the GA at the moment of membranes’ rupture into subgroup 1 (<33 weeks of gestation and 6 days, *n* = 168 patients) and subgroup 2 (at least 34 completed weeks of gestation, *n* = 858 patients). Multiple logistic regressions and interaction analyses adjusted for GA considering five composite adverse neonatal outcomes and predictors were employed. **Results**: PROMs and high c-reactive protein (CRP) values significantly increased the risk of composite outcome 1 occurrence by 14% (95%CI: 1.03–1.57, *p* < 0.001). PROMs and high CRP values increased the risk of composite outcome 5 by 14% (95%CI: 1.07–1.78, *p* < 0.001), PROM and leukocytosis by 11% (95%CI: 1.02–1.59, *p* = 0.001), and PROMs and high PCT values by 21% (95%CI: 1.04–2.10, *p* < 0.001). **Conclusions**: The combination of PROMs and high CRP values significantly increased the risk of all evaluated adverse composite outcomes in early-preterm neonates and should point to careful monitoring of these patients.

## 1. Introduction

Prolonged prelabour rupture of membranes (PROMs) is a medical concept defined as the rupture of membranes for more than 18 h before birth, and it can occur before 37 completed weeks of gestation (preterm prelabour rupture of membranes, PPROMs) or at term, after 37 weeks of gestation [1].

Prolonged ROMs could have a significant impact on the development of adverse neonatal outcomes such as chorioamnionitis followed by the early-onset of neonatal sepsis, bronchopulmonary dysplasia, adverse neurodevelopmental outcomes, and preterm delivery [2,3,4,5]. However, part of the literature data failed to confirm some of the associations between adverse neonatal outcomes and prolonged rupture of membranes, suggesting gestational age at birth to be a significant confounder that was not always taken into consideration [6,7].

For example, a secondary analysis of a randomized controlled trial was conducted to assess the impact of prolonged latency after PPROMs on the occurrence of adverse neurodevelopmental outcomes [8]. The authors established a 3-week cut-off between diagnosis and delivery for group segregation, as well as motor and mental Bayley scores of <70 at two years of age as the primary outcome in a cohort of 1305 patients with PPROMs. Their results indicated that the main outcome was similar between the evaluated groups in the univariate analysis [8]. However, after adjusting for confounders, a prolonged rupture of membranes for more than 3 weeks until delivery was confirmed as an independent risk factor for low motor and mental Bayley scores at the end of the follow-up. Also, gestational age was confirmed as a significant confounder for the occurrence of adverse neurodevelopmental outcomes.

On the other hand, a retrospective study that included 84 infants with PPROMs of more than 48 h analyzed the association between duration of PPROMs and respiratory and neurological outcomes [9]. Their results indicated that prolonged rupture of membranes was not associated with chorioamnionitis or with adverse neurological outcomes even after adjusting for gestational age. However, prolonged rupture of membranes was significantly associated with adverse respiratory outcomes after adjusting for gestational age at birth.

The primary aim of this prospective study was to assess the newborns’ inflammatory marker dynamics in the first 72 h after birth depending on the duration of rupture of membranes (less than 18 h and more than 18 h). The secondary aim of this study was to evaluate the association between prolonged rupture of membranes, newborns’ serum inflammatory markers, and composite adverse neonatal outcomes after adjusting for gestational age at birth.

## 2. Materials and Methods

This prospective cohort study included singleton pregnancies with ruptured membranes (less than 18 h and more than 18 h) who gave birth at “Cuza Voda” Clinical Hospital of Obstetrics and Gynecology, Iasi, Romania, between January 2020 and December 2023. The exclusion criteria comprised the following: maternal age under 18 years old, multiple pregnancies, antenatal diagnosis of major structural abnormalities and/or genetic disorders, previous amniocentesis, mothers with conditions that could have an impact on the fetal or newborn inflammatory response (i.e., autoimmune disorders, viral infections, etc.), incomplete medical records, or inability to provide informed consent.

Ethical approval for conducting this study was obtained from the Institutional Ethics Committees of Grigore T. Popa University of Medicine and Pharmacy, Iasi, Romania (No. 175/17 April 2022) and from Cuza Voda Clinical Hospital of Obstetrics and Gynecology (No. 5751/9 May 2022). All patients signed an informed consent for data processing.

The following type of data was collected from mothers: maternal age, medium of living, degree of antenatal care, parity, the presence of antepartum hemorrhage, fever, or comorbidities, type of medication received (i.e., antibiotics, corticosteroids, etc.), maternal hematological and inflammatory markers (hemoglobin—Hb, white blood cells—WBCs, and C-reactive protein—CRP), results from vaginal, urinary, and amniotic cultures, type of delivery, time from the membranes rupture until delivery, aspect of amniotic fluid, and the confirmation of clinical and/or histopathological chorioamnionitis.

The following types of data were collected from neonates: gestational age at birth, birthweight and Apgar scores (at 1, 5, and 10 min), the need for postpartum resuscitation, WBC count (0–12 h, 24–48 h, and 72–96 h), CRP serum values (0–12 h, 24–48 h, and 72–96 h), immature to total neutrophil—I/T ratio (0–12 h, 24–48 h, and 72–96 h), procalcitonin (0–12 h, 24–48 h, and 72–96 h), as well as adverse neonatal outcomes: metabolic acidosis, hypoglycemia, colonization, digestive intolerance, necrotizing enterocolitis, intraventricular hemorrhage, respiratory distress syndrome, thrombocytopenia, number of days of antibiotic therapy, and death.

A total of 1026 patients and their newborns were included in this study and further segregated into the following groups according to the ACOG criteria [1]: group 1 (rupture of membranes less than 18 h, *n* = 447 patients) and group 2 (rupture of membranes more than 18 h, *n* = 579 patients). These groups were further segregated depending on the gestational age at the moment of the membranes rupture into subgroup 1 (<33 weeks of gestation and 6 days, *n* = 168 patients) and subgroup 2 (at least 34 completed weeks of gestation, *n* = 858 patients).

In the first part of our analysis, we compared the baseline maternal and neonatal characteristics between groups and subgroups using chi-square or Fisher exact tests for categorical variables and t-tests for continuous variables. The results were reported as count number and percentages for categorical variables and as mean and standard deviation for continuous variables. For ordinal variables such as the Apgar score, we used the Mann–Whitney U test (Wilcoxon rank-sum test) for comparisons and reported the medians and interquartile ranges.

In the next step of the analysis, we performed multiple logistic regressions and interaction analyses adjusted for gestational age, considering composite adverse neonatal outcomes and predictors that reunited the terms prolonged rupture of membranes and inflammatory markers values from neonates.

The following composite outcomes were considered: composite_outcome1: Apgar score < 7 at 1 min of birth + respiratory distress syndrome + non-invasive ventilation; composite_outcome2: Apgar score < 7 at 1 min of birth + respiratory distress syndrome + metabolic acidosis; composite_outcome3: small for gestational age + necrotizing enterocolitis + prolonged antibiotic therapy; composite_outcome4: complex reanimation + intraventricular hemorrhage + Apgar score < 7 at 1 min of birth; and composite_outcome5: chorioamnionitis + colonization + early-onset sepsis.

We reported the results as odds ratios (ORs) and 95% confidence intervals (CIs). A *p* value less than 0.05 was considered statistically significant. These analyses were performed using STATA SE (version 17, 2023, StataCorp LLC, College Station, TX, USA).

## 3. Results

The baseline maternal characteristics of 1026 patients are presented in Table 1. Our data indicated that both groups were similar regarding the following characteristics: maternal age at birth (*p* = 0.08), medium of living (*p* = 0.81), degree of monitoring during pregnancy (*p* = 0.15), and parity (*p* = 0.28).

Similar proportions of patients declared vaginal infections at admission (group 1: 7.38% versus group 2: 9.33%, *p* = 0.26), while maternal fever was documented only for three patients (0.53%) in the first group (*p* = 0.12).

Urinary tract infection was detected in 30 patients included in the second group (5.18%) and in 12 patients included in the first group (2.68%), which indicated a significantly higher prevalence of this pathology in patients with prolonged rupture of membranes (*p* = 0.04). The bacterial spectrum of UTIs was similar for both groups and included *Escherichia coli* (group 1: 6 patients, 50% versus group 2: 21 patients, 70%), *Klebsiella* spp. (group 1: 3 patients, 25% versus group 2: 3 patients, 10%), and *Staphylococcus* spp. (group 1: 3 patients, 25% versus group 2: 3 patients, 10%). Also, *Streptococcus* spp. was identified in three urinary cultures (10%) from patients included in the second group.

Patients with prolonged rupture of membranes presented with a significantly higher number of positive amniocultures (141 positive cultures, 24.25%) in comparison with patients who had ruptured membranes for less than 18 h. The microbial spectrum for positive cultures in the first group included *Candida* spp. (7, 13.72%), *Escherichia coli* (9, 17.764%), *Klebsiella* spp. (3, 5.88%), *Enterococcus* spp. (1, 1.96%), *Staphylococcus* spp. (6, 11.76%), *Streptococcus* spp. (12, 23.52%), and *Pseudomonas* spp. (3, 5.88%). Also, the microbial spectrum for positive cultures in the second group comprised *Candida* spp. (39, 27.65%), *Escherichia coli* (46, 32.62%), *Klebsiella* spp. (12, 8.51%), *Enterococcus* spp. (7, 4.96%), *Staphylococcus* spp. (9, 6.38%), *Streptococcus* spp. (15, 10.63%), *Morganella morganii* (3, 2.12%), and other species (10, 7.09%).

Antibiotic treatment received in the previous month before admission was confirmed for 54 patients (12.33%) in the first group and in 204 patients in the second group (35.98%), *p* < 0.001. Additionally, the mean value for maternal CRP was significantly higher in the second group compared to the first group (13.76 ± 24.26 vs. 8.16 ± 13.02, *p* < 0.001).

The baseline neonatal characteristics and outcomes are presented in Table 2. Our results indicated that newborns from mothers who had ruptured membranes for more than 18 h had significantly lower birthweight, gestational age at birth, and Apgar scores at 1, 5, and 10 min (*p* < 0.001) in comparison with newborns from mothers with ruptured membranes for less than 18 h. The gender distribution was relatively similar between the evaluated groups (*p* = 0.17).

Antenatal corticosteroid administration and postpartum resuscitation were significantly more frequently employed in the second group compared to the first group (*p* < 0.001). Also, bacterial colonization of the external auditory canal, pharynx, and skin was significantly more prevalent in the second group (*p* < 0.001).

Metabolic acidosis, ROP, and thrombocytopenia were recorded only in the second group. Additionally, intraventricular hemorrhage (*p* < 0.001), RDS (*p* < 0.001), and hypoglycemia (*p* = 0.003) were significantly more frequently encountered in the second group compared to the first group. Duration of antibiotic therapy was significantly longer for the second group (3.41 ± 1.00 days versus 4.18 ± 2.04 days, *p* < 0.001).

In Table 3 we presented the comparisons between inflammatory markers in newborns considering the following segregation criteria: time since the membranes’ rupture and birth (less than 18 h, more than 18 h), day of measurement, and gestational age at birth (less than 32 completed weeks of gestation, between 32 and 36 weeks + 6 days, and after 37 completed weeks of gestation).

Our data indicated that there was a significant difference between main groups considering the CRP values determined in the first 12 h of life (*p* = 0.007) and in the 24–48 h interval (*p* = 0.002) but not in the 72–96 h interval (*p* = 0.08). Our subgroup analysis revealed that CRP values determined in the first 12 h were significantly higher for the early-preterm neonates (*p* = 0.006) or for newborns with a gestational age higher than 34 weeks of gestation (*p* = 0.01) who were included in the second group in comparison with those included in the first group.

A similar trend for CRP values was observed when determined in the 24–48 h interval for all evaluated subgroups (<33 weeks of gestation and 6 days, *p* = 0.01; after 34 weeks of gestation, *p* = 0.02) that suffered from prolonged rupture of membranes.

The mean values for WBCs and I/T ratio were not significantly different between evaluated groups and subgroups. PCT values were significantly higher for the second group compared with the first group when determined in the first 12 h (*p* = 0.006) and in the 72–96 h of life (*p* < 0.001), but this difference was maintained only for the subgroup of neonates exceeding 34 weeks of gestation with prolonged rupture of membranes (*p* < 0.001) on its third determination.

Table 4 comprises the results from multiple logistic regressions adjusted for gestational age between inflammatory markers and the presence of prolonged rupture of membranes, considering adverse neonatal outcomes. We found out that prolonged rupture of membranes and high CRP values significantly increased the risk of the composite outcome 1 occurrence by 14% (OR: 1.14, 95%CI: 1.03–1.57, *p* < 0.001) in the evaluated cohort. The same combinations increased the risk of composite outcome 2 occurrence by 12% (OR: 1.12, 95%CI: 1.01–1.86, *p* < 0.001), of composite outcome 3 occurrence by 4% (OR: 1.04, 95%CI: 1.03–1.07, *p* = 0.021), and of composite outcome 4 by 2% (OR: 1.02, 95%CI: 1.003–1.09, *p* = 0.022).

Also, the risk of occurrence of composite outcome 5 was significantly increased when considering the combination of prolonged rupture of membranes and high CRP values by 14% (OR: 1.14, 95%CI: 1.07–1.78, *p* < 0.001), the combination of prolonged rupture of membranes and high WBC values by 11% (OR: 1.11, 95%CI: 1.02–1.59, *p* = 0.001), and a combination of prolonged rupture of membranes and high PCT values by 21% (OR: 1.21, 95%CI: 1.04–2.10, *p* < 0.001)

## 4. Discussion

Prolonged rupture of membranes could be associated with adverse neonatal outcomes, especially for early-preterm neonates. Firstly, this study highlighted the newborns’ inflammatory marker dynamics in the first 72 h after birth depending on the duration of rupture of membranes. Our results indicated that CRP values determined in the first 12 h of life and in the 24–48 h interval were significantly higher for the group with prolonged rupture of membranes, and this trend was also observed for the subgroups of early-preterm neonates and neonates with a gestational age exceeding 34 weeks of gestation. On the other hand, PCT values were significantly higher for the second group compared with the first group when determined in the first 12 h and in the 72–96 h of life, but this difference was maintained only for the subgroup of neonates exceeding 34 weeks of gestation, with prolonged rupture of membranes on its third determination.

PCT, a 116-amino-acid precursor of the hormone calcitonin, is produced by thyroid C-cells as a protein triggered by infection [10]. Its levels are significantly increased in numerous bacterial infections, only marginally higher in localized inflammation, and remain low in viral infections and non-specific inflammatory conditions [11]. PCT can be identified in plasma 2 h post-infection, it escalates within 6 to 8 h, and attains a plateau between 20 and 72 h [12], but its values and dynamics can differ depending on gestational age and specific clinical contexts [13]. Serum PCT concentrations increase more swiftly than CRP levels, which peak rapidly before normalizing and do so more swiftly [14].

It may additionally act as a predictive marker for early onset neonatal sepsis [15,16]. In a recent systematic review, published in 2024, that included 69 studies, PCT had a pooled sensitivity of 79% and a specificity of 91% for predicting early onset neonatal sepsis [17]. In cases of severe inflammation or sepsis, circulating levels of PCT rise swiftly. PCT has become increasingly recognized as an effective prognostic and diagnostic marker of inflammation and serves as an ancillary instrument for guiding antibiotic prescriptions or antibiotic discontinuation [18].

NeoPIns trial enrolled 1710 neonates with a gestational age greater than 34 weeks of gestation, and suspicion of early-onset sepsis, out of which 866 neonates were assigned to the experimental arm and 844 to the control arm [19]. In the experimental arm, PCT was measured at regular intervals, and when two consecutive measurements fell within the normal range for the age, discontinuation of antibiotics was made. The results from this trial indicated that the decision of antibiotic discontinuation based on PCT values was superior to standard of care in reducing antibiotic therapy use in the evaluated cohort [19]. However, it is uncertain if discontinuation of antibiotic therapy in early-preterm neonates would be beneficial.

We found a significant difference between groups regarding the gestational age at birth (group 1: 37.97 ± 2.09 gestational weeks versus group 2: 35.82 ± 4.00 gestational weeks, *p* < 0.001). The difference regarding gestational age between groups is explained by the fact that we want to wait as long as possible for preterm neonates with rupture of membranes until delivery (i.e., for administering corticosteroids, for antibiotic treatment in mothers, for reducing the risks associated with prematurity, etc.).

Secondly, we also aimed to evaluate the association between prolonged rupture of membranes, newborns’ serum inflammatory markers, and composite adverse neonatal outcomes in early-preterm neonates (gestational age less than 34 weeks of gestation). Our results indicated that prolonged rupture of membranes and high CRP values significantly increased the risk of the composite outcome 1 occurrence (Apgar score < 7 at 1 min of birth + respiratory distress syndrome + non-invasive ventilation) in the evaluated cohort. The same combination increased the risk of composite outcome 2 (Apgar score < 7 at 1 min of birth + respiratory distress syndrome + metabolic acidosis) occurrence by 12%.

While this combination increased the risk of composite outcomes 3 (small for gestational age + necrotizing enterocolitis + prolonged antibiotic therapy) by 4% and 4 (complex reanimation + intraventricular hemorrhage + Apgar score < 7 at 1 min of birth) by 2%, the clinical effect was minimal. Finally, prolonged rupture of membranes and high CRP, WBCs, and PCT levels increased the risk of composite outcome 5 (chorioamnionitis + colonization + early-onset sepsis) by 14%, 11%, and 21%, respectively.

Literature data suggests higher rates of adverse neonatal outcomes in patients with prolonged rupture of membranes, especially if this event occurs in the preterm population. For example, a recent cohort study by Abebe et al., that included 160 patients, evaluated the risk of adverse maternal and neonatal outcomes of preterm PROMs [20]. The authors indicated a perinatal mortality rate of 206/1000 births. A low Apgar score at 1 min was the most frequent adverse perinatal outcome encountered in the evaluated cohort, followed by early-onset neonatal sepsis, and admission to the intensive care unit [20]. Also cited in the literature, are significantly higher rates of adverse neonatal outcomes such as intraventricular hemorrhage, respiratory distress syndrome, or neurodevelopmental disorders in early-preterm neonates with prolonged rupture of membranes compared to late-preterm neonates [9,21,22].

However, the literature lacks similar data to compare our results, primarily due to the small sample size of other studies and their failure to account for the most significant confounding factor in this scenario: gestational age at birth. Thus, these aspects represent strong points of our study, especially considering the fact that the effects of the inflammatory markers on the occurrence of adverse composite outcomes in this specific category of newborns were evaluated in conjunction with the influence of prolonged rupture of membranes. This particular perspective on the subject may generate specific hypotheses that require additional testing and could potentially influence clinical practice. Another strong point of this study is its prospective design, which allowed careful monitoring of these patients as well as case documentation, which led to a low rate of missing values (2%), an aspect that can often impose difficulties in data analysis.

However, these findings should be interpreted considering the following limitations: a small follow-up period, a lack of determination of PCT from the umbilical cord blood, which could be potentially more reliable for predicting intraamniotic infection or early onset sepsis, and the patients’ recruitment from a single tertiary center.

Further studies, on larger cohorts of patients, should test in a prospective setting the influence of multiple panels of inflammatory markers determined from the newborn’s serum on the occurrence of adverse neonatal outcomes.

## 5. Conclusions

CRP values determined in the first two days of life were significantly higher for the group with prolonged rupture of membranes, and this trend was maintained regardless of the evaluated gestational age category.

The group with prolonged rupture of membranes had significantly higher PCT values in the first 12 h and 72–96 h of life than the first group. Neonates over 34 weeks of gestation, with prolonged membrane rupture, also had a significantly higher third determination of PCT in comparison with their counterparts.

Even though PCT is cited as a prognostic marker for adverse neonatal outcomes, our results indicated that its high serum levels significantly increased the risk of occurrence of a composite outcome that comprised chorioamnionitis, newborn colonization, and early-onset sepsis.

On the other hand, CRP values were found to significantly increase the risk of all evaluated composite outcomes, and the calculated effect could have a clinical significance only on the occurrence of composite outcome 1 (Apgar score < 7 at 1 min of birth + respiratory distress syndrome + non-invasive ventilation) and 2 (Apgar score < 7 at 1 min of birth + respiratory distress syndrome + metabolic acidosis).

## Figures and Tables

**Table 1:** Baseline maternal characteristics.

Characteristics	Group 1 (Rupture of Membranes for Less Than 18 h, *n* = 447 Patients)	Group 2 (Rupture of Membranes for More Than 18 h, *n* = 579 Patients)	*p* Value
Maternal age at birth, years (mean and standard deviation)	27.71 ± 6.10	28.41 ± 6.63	0.08
Medium of living (*n*/%)	Rural = 270 (60.40%) Urban = 177 (39.60%)	Rural = 354 (61.14%) Urban = 225(38.86%)	0.81
Monitoring during pregnancy (*n*/%)	Complete monitoring = 348 (77.85%) Partially monitored = 42 (9.40%) Not monitored = 57 (12.75%)	Complete monitoring = 426 (73.58%) Partially monitored = 99 (17.10%) Not monitored = 54 (9.33%)	0.15
Parity (*n*/%)	Nulliparous = 282 (63.09%) Multiparous = 165 (36.91%)	Nulliparous = 384 (66.32%) Multiparous = 195 (33.68%)	0.28
Maternal fever (*n*/%)	Yes = 0 (0%)	Yes = 3 (0.53%)	0.12
Known vaginal infection at admission (*n*/%)	Yes = 33 (7.38%)	Yes = 54 (9.33%)	0.26
Urinary tract infection (*n*/%)	Yes = 12 (2.68%)	Yes = 30 (5.18%)	0.04
Positive amnioculture (*n*/%)	Yes = 51 (11.41%)	Yes = 141 (24.35%)	<0.001
Antepartum hemorrhage (*n*/%)	Yes = 6 (1.37%)	Yes = 9 (1.59%)	0.77
Previous antibiotic therapy in the last month (*n*/%)	Yes = 54 (12.33%)	Yes = 204 (35.98%)	<0.001
Hb at admission (mean and standard deviation)	10.82 ± 1.39	10.92 ± 1.26	0.24
WBCs at admission (mean and standard deviation)	12.22 ± 3.56	12.05 ± 3.49	0.45
CRP at admission (mean and standard deviation)	8.16 ± 13.02	13.76 ± 24.26	<0.001

Legend: Hb—hemoglobin; WBCs—white blood cells; CRP—C-reactive protein.

**Table 2:** Baseline neonatal characteristics and outcomes.

Characteristics	Group 1 (Rupture of Membranes for Less Than 18 h, *n* = 447 Patients)	Group 2 (Rupture of Membranes for More Than 18 h, *n* = 579 Patients)	*p* Value
Birthweight, g (mean and standard deviation)	3153.289 ± 564.10	2688.212 ± 949.12	<0.001
GA at birth, weeks (mean and standard deviation)	37.97 ± 2.09	35.82 ± 4.00	<0.001
Sex (*n*/%)	Female = 195 (43.62%) Male = 252 (56.38%)	Female = 228 (39.38%) Male = 351 (60.62%)	0.17
Apgar score at 1 min (median and interquartile range)	9 (8–9)	8 (7–9)	<0.001
Apgar score at 5 min (median and interquartile range)	9 (8–9)	8 (7–9)	<0.001
Apgar score at 10 min (median and interquartile range)	9 (8–9)	8 (8–9)	<0.001
Antenatal corticosteroids (*n*/%)	Yes = 21 (4.70%)	Yes = 96 (16.58%)	<0.001
Need for postpartum resuscitation (*n*/%)	Yes = 6 (1.34%)	Yes = 54 (9.33%)	<0.001
Colonization (*n*/%)	Yes = 3 (0.67%)	Yes = 33 (5.70%)	<0.001
Digestive intolerance (*n*/%)	Yes = 3 (0.67%)	Yes = 3 (0.52%)	0.75
Necrotizing enterocolitis (*n*/%)	Yes = 0 (0%)	Yes = 3 (0.5%)	0.13
Metabolic acidosis (*n*/%)	Yes = 0 (0%)	Yes = 15 (2.59%)	0.001
Intraventricular hemorrhage (*n*/%)	Yes = 18 (4.02%)	Yes = 78 (13.47%)	<0.001
ROP (*n*/%)	Yes = 0 (0%)	Yes = 15 (2.59%)	0.03
RDS (*n*/%)	Yes = 39 (8.72%)	Yes = 162 (27.98%)	<0.001
Early-onset sepsis (*n*/%)	Yes = 6 (1.34%)	Yes = 12 (2.07%)	0.37
Hypoglycemia (*n*/%)	Yes = 12 (2.68%)	Yes = 39 (6.74%)	0.003
Thrombocytopenia (*n*/%)	Yes = 0 (0%)	Yes = 6 (1.04%)	0.03
Days of antibiotic therapy (mean and standard deviation)	3.41 ± 1.00	4.18 ± 2.04	<0.001
Neonatal death (*n*/%)	0 (0%)	9 (1.55%)	0.008

Legend: GA—gestational age; ROP—retinopathy of prematurity; RDS—respiratory distress syndrome.

**Table 3:** Comparisons of inflammatory markers determined in dynamics between evaluated groups and subgroups.

Marker	Group 1	Group 2	*p* Value
CRP 0–12 h	4.93 ± 0.66	9.73 ± 9.10	0.007
CRP 24–48 h	5.66 ± 4.97	8.34 ± 9.23	0.002
CRP 72–96 h	7.25 ± 5.86	8.23 ± 7.86	0.08
CRP 0–12 h < 33W + 6D	5.7 ± 1.32	9.87 ± 10.01	0.006
CRP 0–12 h > 34W	5.69 ± 4.15	9.56 ± 9.84	0.01
CRP 24–48 h < 33W + 6D	4.6 ± 1.32	10.10 ± 7.15	0.01
CRP 24–48 h > 34W	4.52 ± 3.91	8.82 ± 11.87	0.02
CRP 72–96 h < 33W + 6D	4.5 ± 1.36	9.23 ± 10.09	0.64
CRP 72–96 h > 34W	6.76 ± 6.35	6.63 ± 3.93	0.88
WBCs 0–12 h	8.166 ± 2.650	7.611 ± 4.103	0.25
WBCs 24–48 h	15.342 ± 5.714	14.662 ± 6.012	0.30
WBCs 72–96 h	17.082 ± 5.899	17.836 ± 6.983	0.16
WBCs 0–12 h < 33W + 6D	11.200 ± 8.420	17.031 ± 3.903	0.68
WBCs 0–12 h > 34W	22.135 ± 5.068	23.518 ± 6.013	0.08
WBCs 24–48 h < 33W + 6D	17.305 ± 13.881	20.459 ± 15.447	0.39
WBCs 24–48 h > 34W	14.640 ± 4.187	14.197 ± 4.764	0.61
WBCs 72–96 h < 33W + 6D	16.320 ± 13.106	15.343 ± 12.788	0.49
WBCs 72–96 h > 34W	12.150 ± 3.504	12.585 ± 4.633	0.59
PCT 0–12 h	4.42 ± 11.10	8.61 ± 10.64	0.006
PCT 24–48 h	2.08 ± 3.60	3.35 ± 7.92	0.87
PCT 72–96 h	0.41 ± 0.59	2.90 ± 1.06	<0.001
PCT 0–12 h < 33W + 6D	1.22 ± 4.14	1.46 ± 8.22	0.70
PCT 0–12 h > 34W	2.11 ± 4.04	2.44 ± 8.35	0.67
PCT 24–48 h < 33W + 6D	1.41 ± 1.32	2.95 ± 0.65	0.46
PCT 24–48 h > 34W	1.07 ± 1.72	1.73 ± 1.91	0.43
PCT 72–96 h < 33W + 6D	0.23 ± 1.06	1.35 ± 0.09	0.67
PCT 72–96 h > 34W	1.05 ± 0.08	1.43 ± 0.83	<0.001
I/T 0–12 h	0.09 ± 0.07	0.18 ± 0.05	0.26
I/T 24–48 h	0.05 ± 0.01	0.14 ± 0.08	0.25
I/T 72–96 h	0.03 ± 0.09	0.06 ± 0.04	0.77
I/T 0–12 h < 33W + 6D	0.12 ± 0.08	0.16 ± 0.02	0.72
I/T 0–12 h >34W	0.11 ± 0.04	0.14 ± 0.02	0.88
I/T 24–48 h < 33W + 6D	0.06 ± 0.02	0.15 ± 0.01	0.48
I/T 24–48 h > 34W	0.05 ± 0.06	0.13 ± 0.07	0.54
I/T 72–96 h < 33W + 6D	0.05 ± 0.02	0.06 ± 0.03	0.97
I/T 72–96 h > 34W	0.04 ± 0.08	0.05 ± 0.04	0.92

Legend: WBCs—white blood cells; CRP—C-reactive protein; PCT—procalcitonin; I/T—immature to total neutrophil ratio; W—weeks; D—days.

**Table 4:** Results from multiple logistic regressions and interaction analyses between inflammatory markers and the presence of prolonged rupture of membranes, considering adverse neonatal outcomes in early-preterm neonates.

Composite Outcome	Predictor	Odds Ratio	95% Confidence Interval	*p* Value
CO_1	Group 2	1.35	0.59–3.11	0.469
	Gestational age	0.49	0.38–1.64	<0.001
	CRP values	1.04	1.01–1.07	0.004
	WBC values	0.97	0.93–1.01	0.171
	PCT values	0.98	0.90–1.06	0.713
	Group2##CRP *	1.14	1.03–1.57	<0.001
	Group2##WBCs *	1.02	0.99–1.05	0.233
	Group2##PCT *	0.98	0.95–1.01	0.210
CO_2	Group 2	3.64	1.72–7.67	0.001
	Gestational age	0.44	0.34–0.57	<0.001
	CRP values	1.05	1.01–1.08	0.002
	WBC values	1.004	0.95–1.05	0.866
	PCT values	0.93	0.84–1.07	0.426
	Group2##CRP *	1.12	1.01–1.86	0.003
	Group2##WBCs *	0.87	0.70–1.02	0.101
	Group2##PCT *	0.98	0.95–1.01	0.210
CO_3	Group 2	2.66	1.08–6.55	0.033
	Gestational age	0.20	0.10–0.38	<0.001
	CRP values	1.04	1.01–1.07	0.004
	WBC values	0.97	0.93–1.01	0.171
	PCT values	0.98	0.90–1.06	0.713
	Group2##CRP *	1.04	1.01–1.07	0.021
	Group2##WBCs *	1.004	0.98–1.03	0.775
	Group2##PCT *	0.99	0.96–1.01	0.325
CO_4	Group 2	2.79	1.29–6.00	0.009
	Gestational age	0.49	0.38–0.63	<0.001
	CRP values	1.04	1.00–1.07	0.014
	WBC values	0.99	0.94–1.04	0.723
	PCT values	0.95	0.85–1.06	0.382
	Group2##CRP *	1.02	1.003–1.09	0.022
	Group2##WBCs *	1.01	0.99–0.05	0.215
	Group2##PCT *	1.04	0.93–1.16	0.427
CO_5	Group 2	3.70	1.46–9.40	0.006
	Gestational age	0.86	0.77–0.96	0.008
	CRP values	1.06	1.03–1.10	<0.001
	WBC values	1.04	1.01–1.06	0.001
	PCT values	1.07	1.02–1.11	<0.001
	Group2##CRP *	1.14	1.07–1.78	<0.001
	Group2##WBCs *	1.11	1.02–1.59	0.001
	Group2##PCT *	1.21	1.04–2.10	<0.001

Legend: WBCs—white blood cells; CRP—C-reactive protein; PCT—procalcitonin; RDS—respiratory distress syndrome; CO_1 (composite outcome1): Apgar score < 7 at 1 min of birth + respiratory distress syndrome + non-invasive ventilation; CO_2 (composite_outcome2): Apgar score < 7 at 1 min of birth + respiratory distress syndrome + metabolic acidosis; CO_3 (composite_outcome3): small for gestational age + necrotizing enterocolitis + prolonged antibiotic therapy; CO_4 (composite_outcome4): complex reanimation + intraventricular hemorrhage + Apgar score < 7 at 1 min of birth; CO_5 (composite_outcome5): chorioamnionitis + colonization + early-onset sepsis. *—adjusted for gestational age.

## Data Availability

The data presented in this study are available upon request from the corresponding author. The data are not publicly available due to local policies.

## References

[1] American College of Obstetricians and Gynecologists (2020). Prelabor Rupture of Membranes: ACOG Practice Bulletin, Number 217. Obstet. Gynecol..

[2] Hincu M.A., Zonda G.I., Stanciu G.D., Nemescu D., Paduraru L. (2020). Relevance of Biomarkers Currently in Use or Research for Practical Diagnosis Approach of Neonatal Early-Onset Sepsis. Children.

[3] Hincu M.A., Zonda G.I., Vicoveanu P., Harabor V., Harabor A., Carauleanu A., Melinte-Popescu A.S., Melinte-Popescu M., Mihalceanu E., Stuparu-Cretu M. (2024). Investigating the Association between Serum and Hematological Biomarkers and Neonatal Sepsis in Newborns with Premature Rupture of Membranes: A Retrospective Study. Children.

[4] Park J.H., Bae J.G., Chang Y.S. (2021). Neonatal Outcomes according to the Latent Period from Membrane Rupture to Delivery among Extremely Preterm Infants Exposed to Preterm Premature Rupture of Membrane: A Nationwide Cohort Study. J. Korean Med. Sci..

[5] Walker M.W., Picklesimer A.H., Clark R.H., Spitzer A.R., Garite T.J. (2014). Impact of duration of rupture of membranes on outcomes of premature infants. J. Perinatol..

[6] Madan I., Jackson F.I., Figueroa R., Bahado-Singh R. (2023). Preterm prelabor rupture of membranes in singletons: Maternal and neonatal outcomes. J. Perinat. Med..

[7] Park G.Y., Park W.S., Sung S.I., Kim M.S., Lee M.H., Jeon G.W., Kim S.S., Chang Y.S. (2022). Neonatal outcome comparisons between preterm infants with or without early pulmonary hypertension following prolonged preterm premature rupture of membranes before 25 gestational weeks in Korean Neonatal Network. J. Matern. Fetal Neonatal Med..

[8] Drassinower D., Friedman A.M., Običan S.G., Levin H., Gyamfi-Bannerman C. (2016). Prolonged latency of preterm prelabour rupture of membranes and neurodevelopmental outcomes: A secondary analysis. Bjog.

[9] Müller H., Stähling A.C., Bruns N., Weiss C., Ai M., Köninger A., Felderhoff-Müser U. (2022). Latency duration of preterm premature rupture of membranes and neonatal outcome: A retrospective single-center experience. Eur. J. Pediatr..

[10] Davies J. (2015). Procalcitonin. J. Clin. Pathol..

[11] Go H., Nagano N., Katayama D., Akimoto T., Imaizumi T., Aoki R., Hijikata M., Seimiya A., Kato R., Okahashi A. (2020). Diagnostic Accuracy of Biomarkers for Early-Onset Neonatal Bacterial Infections: Evaluation of Serum Procalcitonin Reference Curves. Diagnostics.

[12] Hu Y., Yang M., Zhou Y., Ding Y., Xiang Z., Yu L. (2017). Establishment of reference intervals for procalcitonin in healthy pregnant women of Chinese population. Clin. Biochem..

[13] Tuoni C., Ciantelli M., Morganti R., Violi M., Tamagnini S., Filippi L. (2022). Procalcitonin levels in preterm newborns: Reference ranges during the first three days of life. Front. Pediatr..

[14] Meisner M. (2002). Pathobiochemistry and clinical use of procalcitonin. Clin. Chim. Acta.

[15] Eschborn S., Weitkamp J.H. (2019). Procalcitonin versus C-reactive protein: Review of kinetics and performance for diagnosis of neonatal sepsis. J. Perinatol..

[16] Ruan L., Chen G.Y., Liu Z., Zhao Y., Xu G.Y., Li S.F., Li C.N., Chen L.S., Tao Z. (2018). The combination of procalcitonin and C-reactive protein or presepsin alone improves the accuracy of diagnosis of neonatal sepsis: A meta-analysis and systematic review. Crit. Care.

[17] van Leeuwen L.M., Fourie E., van den Brink G., Bekker V., van Houten M.A. (2024). Diagnostic value of maternal, cord blood and neonatal biomarkers for early-onset sepsis: A systematic review and meta-analysis. Clin. Microbiol. Infect..

[18] Chambliss A.B., Patel K., Colón-Franco J.M., Hayden J., Katz S.E., Minejima E., Woodworth A. (2023). AACC Guidance Document on the Clinical Use of Procalcitonin. J. Appl. Lab. Med..

[19] Stocker M., van Herk W., El Helou S., Dutta S., Fontana M.S., Schuerman F., van den Tooren-de Groot R.K., Wieringa J.W., Janota J., van der Meer-Kappelle L.H. (2017). Procalcitonin-guided decision making for duration of antibiotic therapy in neonates with suspected early-onset sepsis: A multicentre, randomised controlled trial (NeoPIns). Lancet.

[20] Abebe T., Nima D., Mariye Y., Leminie A. (2024). The magnitude of maternal and neonatal adverse outcomes associated with preterm premature rupture of membrane: A prospective cohort study. J. Neonatal Nurs..

[21] Giamberardino W., Winn V.D., Armstrong J. (2021). Risk factors for clinically significant intra-ventricular hemorrhage in pregnancies complicated by preterm premature rupture of membranes. J. Pregnancy Neonatal Med..

[22] Mishra S., Jaiswar S., Saad S., Tripathi S., Singh N., Deo S., Agarwal M., Mishra N. (2021). Platelet indices as a predictive marker in neonatal sepsis and respiratory distress in preterm prelabor rupture of membranes. Int. J. Hematol..

